# Departmental conditions for professional learning of instructors in vocational and professional education

**DOI:** 10.1186/s40461-023-00151-z

**Published:** 2023-10-27

**Authors:** Annemarieke Hoekstra

**Affiliations:** Department of Academic Excellence, Northern Alberta Institute Technology, 11762 106 Street NW, Edmonton, AB T5G 2R1 Canada

**Keywords:** Professional learning, Workplace learning, Vocational education and training, Professional education, Instructor learning, Conditions for learning, Informal learning

## Abstract

**Background:**

For vocational and professional education to remain relevant, instructors need to keep developing themselves and their practices. Much of instructor learning happens on-the-job. Drawing on literature on teacher learning in the workplace, this article explores how structural and cultural conditions shape professional learning of instructors in departments for post-secondary vocational and professional education in western Canada.

**Methods:**

A multiple case study approach was used to explore how instructors perceive departmental conditions as enhancing or inhibiting professional learning. Interview data, meeting observations, and program documentation was collected from 27 instructors from 5 departments in three institutes for post-secondary vocational and professional education. The educational programming in the five departments cover four industry sectors: two healthcare departments, one building trades, one business, and one social services department.

**Results:**

Structural conditions reported to facilitate instructor professional learning at the department level include student feedback, job-rotation, coordinating work-placements, and whether participation in continuing professional development is a licensing requirement of the profession. Heavy workload and the way teaching is scheduled are most often reported as conditions inhibiting learning. Considering cultural conditions, three in-depth case descriptions illustrate how instructors draw on beliefs and practices prevalent in their original trade/profession when shaping their departmental culture as a learning environment.

**Conclusions:**

The concept of sense-making proved useful to describe how instructors draw on elements of the occupational culture taught in the program when shaping their workplace as a learning environment. This influence of occupational culture could help explain previously observed differences in how instructors from various industry sectors engage in professional learning. Organizational support is warranted for facilitating organizational conditions for instructor learning including the development of departmental leaders’ capacity to influence workplace conditions for professional learning.

**Supplementary Information:**

The online version contains supplementary material available at 10.1186/s40461-023-00151-z.

## Introduction

Increasingly rapid changes in society and industry necessitate ongoing learning by instructors in post-secondary public vocational and professional education (VPE) (Andersson and Köpsén 2017; Darwin 2007). Such ongoing instructor learning happens on-the-job, and is mostly informal, tacit, and gained through practical work experience (Viskovic 2005; Hoekstra et al. 2018). It would follow that organizations could foster such ongoing instructor learning by optimizing workplace conditions for learning. Yet, research on the impact of workplace conditions in VPE on learning of VPE instructors is limited (Viskovic 2005; Bound 2011). Hence, our study explores how conditions in five departments in three VPE institutes in western Canada impact on the learning of instructors in those departments.

The last two decades have seen a burgeoning of studies into organizational conditions affecting workplace learning in general (Kyndt and Baert 2013), and informal learning by elementary and secondary school teachers specifically (Kyndt et al. 2016). Frequently identified conditions affecting workplace learning of elementary and secondary school teachers include: autonomy, job variety and workload, organizational support, time, school culture, collaboration opportunities, leadership, and peer support. It is unclear, however, whether these workplace conditions would similarly impact on workplace learning of instructors in institutes for VPE.

A few recent studies have focused on factors promoting professional learning in VPE. In a study on the relationship between transformational leadership and social learning activities of teachers in vocational education and training (VET), Oude Groote Beverborg et al. (2015) found that VET teachers do not distinguish between asking for feedback and sharing information. The authors suggest that future research could investigate whether differences in social learning could be explained by differences in environmental factors or differences in personal factors. Andersson and Köpsén (2015) found that vocational teachers from different industry sectors differed in the extent to which they participated in continuing professional development (CPD) opportunities, specifically they found lower participation rates from the Technology sector, as compared to Services and Vehicles sector. The authors hypothesize that possibly educational background and beliefs about their ability to successfully participate in industry practices as a form of CPD, may play a role in varying CPD participation rates across industry sectors (Andersson and Köpsén 2015, p. 17).

Circumstances VPE instructors find themselves in, at least in Western Canada, suggest that conditions for workplace learning may impact on instructor learning in different ways than conditions in elementary and secondary schools on teachers. These circumstances include the need for (1) a dual professionalism and (2) the complex organizational structure of institutes for VPE. The following sections elaborate on each of these.

### VPE instructor dual professionalism in a Western Canadian context

Vocational and Professional Education (VPE) in Western Canada encompasses both trades education (e.g. carpenter, electrician, pipefitter) as well as degree studies that prepare for a specific job, such as nursing or social work (Anderson 2008). As such, VPE includes but is not limited to what in Europe is commonly referred to as vocational education and training (VET) and technical and vocational education and training (TVET) (Tripney and Hombrados 2013).

Those who teach in Canadian institutes for post-secondary VPE are called instructors, or occasionally professors. In this paper we use the more common term instructor to describe anyone who teaches in a Canadian post-secondary VPE institute. Education is governed provincially. While instructors are expected to be licensed in the trade/profession they teach, Canadian provinces do not require post-secondary instructors to hold teaching credentials (Hoekstra and Crocker 2015; Viskovic 2005). Instructors teach theory classes and where applicable they teach labs and/or shop classes. While some instructors have a bachelor’s degree in education and are licenced as teachers by the province, most do not. Unlike UK and European vocational education systems, there is no distinction in duties, tasks, or salary between instructors with a teacher’s license and instructors without that license. Beginning instructors are fully employed as instructors from day one of their first employment at the post-secondary VPE institute. Not only do instructors need to learn how to teach when they are already on-the-job teaching, they also need to stay abreast of developments in their trade or profession (Andersson and Köpsén 2017).

Thus, unlike teachers in elementary and secondary schools, VPE instructors need to stay connected to the trade or industry profession they teach, develop and maintain their own curriculum, while also developing themselves as educators. Consequently, instructors in VPE in Western Canada need to seek out learning opportunities to develop both educational (how to create a good grading rubric) as well as industry competencies (e.g. how to repair electronic vehicles).

The curriculum of diploma and degree programs in VPE in Western Canada is the responsibility of the post-secondary institute. The curriculum is developed by program instructors with input from a program advisory committee of professionals from that field (e.g. accountants, nurses) and the professional association affiliated with their diploma/degree. Seeking this input provides a way for instructors to stay current with developments in the field. Another possible workplace condition that could aid instructors in staying up to date with industry standards includes collaboration with the industry professionals who serve as on-the-job practicum supervisors of students.

The curriculum in trades programs is created and mandated by the provincial government. Trade students will work about 9–10 months per year in the trade, then come to the VPE institute for 2–3 months per year. The post-secondary institutes have workshops and labs for each trade. For each trade the provincial government provides a list of learning objectives, hours to be spent on each learning objective, as well as all the written course content organized in a set of learning modules by learning objective per course. Trade students buy these learning modules and use them as their main study material. Instructors present and explain the content during theory classes. They also provide opportunities for skill development during shop classes.

### Department level practices in institutes for VPE in a Western Canadian context

Instructors in VPE in Western Canada are organized in departments. Typically, each department is responsible for one program, or occasionally 2–3 related smaller programs. As each VPE department is responsible for ensuring that its curriculum and program delivery optimally prepare students for current industry practices, it can be expected that instructors experience shared goals at the level of the department, rather than the level of the whole school as in elementary schools (e.g. Louis et al., 1996). Additionally, each department is led by a department chair who is an instructor from the program on a temporary chair assignment. The chair is responsible for budgeting, scheduling, supervision of instructors, and the leadership of the curriculum and instructors in the program. In their study on transformational leadership in TVET institutes, Oude Groote Beverborg and colleagues (2015) hypothesize that, in contrast to studies conducted in elementary and secondary schools, the complexity of the organizational environment of the TVET institution might decrease the effect of institutional transformational leadership on professional learning of instructors, due to multiple levels of organization, units, and subunits in post-secondary contexts. Indeed, a previous study highlighted the important role department chairs play in fostering instructor learning (Hoekstra et al. 2018).

Secondly, the instructors in each department have in common that they each were certified tradespeople and industry professionals in the same trade/profession prior to becoming an instructor. VPE departments thus each also represent the professionalism from the trade/profession taught in the department.

### Present study

To summarize, there is an urgent need for VPE instructors to stay current as industry professional and educator, necessitating ongoing professional learning. To understand how to foster such learning, insight into workplace conditions impacting said learning is required. While research into workplace conditions for teacher learning in elementary and secondary school could provide some of this insight, it is unclear whether these workplace conditions would similarly impact on workplace learning of instructors in institutes for VPE, due to two circumstances in VPE that are different: 1.) VPE instructors’ need to maintain a dual professionalism, and 2.) the organizational structure of institutes for VPE is complex and nested. Hence, further exploration of department level conditions for instructor learning in VPE is warranted. Additionally, some findings suggest that instructors from different industry sectors may differ in the extent to which they engage in continuous professional development. By including departments from various industry sectors (building trades, business, healthcare, social services) we aim to further explore potential differences. Hence, our study focuses on workplace conditions for professional learning of 27 VPE instructors across 5 VPE departments in three VPE institutes.

## Conceptual framework

### Professional learning

The present study is grounded in a socio-cultural perspective on practice and learning (Lave and Wenger 1991; Wenger 1998). Research on professional learning shows that it is deeply embedded in practice and is informed by the way people conduct and understand their work (Bound 2011; Engeström 2011; Lave and Wenger 1991). Most conceptualizations of learning imply a relatively lasting change in behaviour or capacity for behaviour (Fraser et al. 2007). The capacity for behaviour refers to the knowledge, skills and/or attitudes that enable the learner to demonstrate certain behaviour (Stes et al. 2010). The present study defines professional learning as engaging in activities which lead to changes in professional practice or the capacity to make changes to professional practice (Webster-Wright 2009; Opfer and Pedder 2011).

One of the central tenets of a socio-cultural perspective on learning is that knowledge and skills are *situated* in practice (Billett 2004). Billett (2004) describes that groups of people, or communities, engage in similar practice usually with the goal to sustain that practice. The learning of members of the community is situated in the activities and practices undertaken by the community. It is thus that members of the community ‘learn by being part of a social context of real practice’ (Viscovic 2005, p. 392). Lave and Wenger (1991) describe that through participation in the practice of the community (i.e. the work that is carried out by the community) members of the community develop knowledge and experience and gain more legitimacy and responsibility in the community, deriving their professional identity from being full members of that community (Lave and Wenger 1991). Andersson and Köpsén (2015) explain that to be knowledgeable enough to teach a trade/profession, VPE instructors must have “developed an identity of full membership and participation in a specific community of practice” (p. 4). For instance, to be able to teach nursing, a VPE instructor must be a recognized member of the community of practice that is the nursing profession. Once hired as a post-secondary instructor VPE instructors become part of a second community of practice: the practice of the community that is post-secondary education. Within this context by participating in the activities of their instructional department they develop a second professionalism as educator. Without the requirement to obtain a teaching credential, VPE instructors develop their instructional knowledge and skills largely by doing: by participating in the work of the educational program they teach in (Hoekstra and Newton 2017; Viscovic 2005). As this learning happens primarily on-the-job, it is important to consider what and how workplace practices foster such learning. The next section further theorizes how workplace practices shape conditions for professional learning.

### Conditions for professional learning

Conditions for professional learning arise from the way everyday work tasks are shaped, including opportunities for collaboration and feedback, and resources such as time and interaction (Skule and Reichborn 2002, 10; Billett 2004). In line with this literature, we define workplace conditions as the specific work-related circumstances instructors find themselves in that either create or inhibit opportunities for learning.

Quite a few studies have focused on identifying workplace conditions that optimize teacher learning at work (Kyndt et al. 2016). Recommendations include suggestions for optimizing workplace learning by creating conditions favourable for such learning. However, studies have shown that improving workplace conditions for learning does not guarantee enhanced performance, as the impact of workplace conditions on learning is mediated by teachers’ perceptions and interpretations of these conditions (Hoekstra et al. 2009; Louws et al. 2017). Billett (2004) argues in this respect that “situational factors alone are insufficient to understand workplaces as learning environments.” He continues: “What is required is an understanding of the way individuals’ agentic action and intentionalities shape how they participate in and learn from work.” (p. 316). Moreover, individuals’ agentic actions do not occur in isolation, people make sense of their surroundings through interaction, drawing on ways of thinking available to them. In a study into how teachers implement policy, Coburn (2006) used sense-making theory to explain teachers’ interactions and practices. Sense-making theory purports that people undertake action based on the information they select from their environment and how they make meaning of that information. This meaning making or sense-making is social in two respects. First, it is collective, in that it is shaped in interaction: people talk with each other to create a shared understanding. Second, it is situated: “individuals and groups draw on ideas or approaches available to them in their proximal communities as they make sense of their situation: larger systems of beliefs, elements of occupational culture, and organization- or workgroup-specific premises or traditions” (Coburn 2006, p. 345). Thus, to further understand how workplace conditions affect ongoing professional learning of VPE instructors, this study explores how instructors’ own perceptions and interpretations of their work and learning inform their actions and help shape the workplace as a learning environment.

To inform our study into workplace conditions for professional learning, we draw on insights from literature on teacher workplace learning. This literature distinguishes structural and cultural conditions within the workplace (Kyndt et al. 2016; Louws et al. 2017). Each is described in more detail below.

### Structural conditions

According to Louws et al. (2017), “structural conditions refer to the way schools, teachers’ work, and teachers’ learning are organized structurally in terms of time, space, resources, … evaluation and feedback, organizational goals, and professional development policies” (p.4). Structural elements include the way classes are scheduled, institutional administration of student evaluation, and the physical location of classrooms and workspaces on campus. Typically, instructors are responsible to teach their own classes. Lohman (2006) observed that teaching classes individually is not naturally conducive to learning through collaboration and peer observation (Lohman 2006), as would be the case in professions where much of the work happens in pairs or in teams, such as policing or nursing. When collaboration does not naturally occur, shared office space and a common room are appreciated spaces for informal learning (Kyndt et al. 2016). As opportunities for working alongside colleagues are limited, peer feedback does not naturally occur and needs to be actively sought out by instructors inviting each other into their classrooms. Student feedback on the other hand is more readily available, especially when institutions employ student feedback survey systems.

A second set of structural conditions that have been related to teacher learning involve organizational provisions that promote learning and enhance performance, such as funding and resources for professional development, internet access (Lohman 2006), performance management policies and procedures (Runhaar and Sanders 2016), and social recognition for learning and development (Kyndt et al. 2016). Finally, monetary reward, which in many professions is considered a motivator for learning aimed at enhancing performance, is not typically found to motivate teacher professional learning (Mintrop et al. 2018). In the present study, structural conditions within departments for VPE are studied within the context of the larger VPE institute.

### Cultural conditions

School culture refers to “the beliefs, values, habits and assumed ways of doing things among communities of teachers who have had to deal with similar demands and constraints over many years” (Hargreaves 1994, 165). When teachers make sense of teaching and themselves as a teacher, the interpretations they make are mediated by cultural tools (Coburn 2006) and bound by “cognitive frameworks and affective templates as well as institutional practices” (Coldron and Smith 1999, 715) that exist in the social space in which they work. Culture thus encompasses both what instructors believe about teaching and learning, as well as how instructors learn from each other (Viskovic 2005).

To explore cultural conditions as they exist at the departmental level, we considered those conditions that are part of the socio-cultural practices in instructors’ daily work environments (Hoekstra et al. 2009) rather than macro-organizational factors. From the literature on teacher learning, conditions that come most clearly to the fore as cultural aspects that inform teacher learning in their daily teaching practice (Imants et al. 2013) include:

1. Peer collaboration and interaction
2. Shared norms and responsibilities
3. Autonomy
4. Perceptions of departmental leadership

While this list of conditions may not be exhaustive, for the purpose of the present study these four conditions serve as a guide to explore the socio-cultural practices that shape conditions for learning in instructors’ daily work environment.

#### Peer collaboration and interaction

Large scale review studies on teacher learning describe that many studies have demonstrated the importance of collaboration for teacher learning (Opfer and Pedder 2011; Kyndt et al. 2016). Through collaboration teachers exchange ideas, engage in reflective discussions, develop new materials, and provide moral support (Meirink et al. 2010; Hoekstra et al. 2009). Simply increasing collaboration does not, however, necessarily enhance teacher learning (Opfer and Pedder 2011, p. 386).

Little (1990) distinguished levels of interdependence within teacher collaboration. Interdependence refers to the extent to which teachers depend on each other for the successful completion of their tasks; The higher the level of interdependence, the richer the collaboration becomes in offering opportunities for professional learning (Little 1990; Meirink et al. 2010). Examples of collaboration with low levels of interdependence include storytelling and experience swapping. In these situations, teachers informally exchange stories of their experiences in the classroom, without expectation that teachers change their practices. A moderate level of interdependence exists in situations where teachers exchange instructional materials and ideas. In these situations, teachers may act upon the advice received. A high level of interdependence includes joint work, where more than one teacher’s expertise is needed for problem solving (Clement and Vandenberghe 2000; Little 1990; Meirink et al. 2010). An example of joint work is the creation of program level assessments, which affects the collective practice of the group and necessitates explicating beliefs and negotiating meaning and goals, providing a rich environment for professional learning. In the present paper, collaboration as a condition for learning is studied in terms of opportunities that exist in the workplace for instructors to interact and collaborate at various levels of interdependence.

#### Shared norms and responsibility

Literature on teacher learning in schools, points to the importance of shared norms and responsibility for teachers to know where they are and to know where they need to go (Rosenholtz 1989). Little (1990) states that “strong professional communities exert collective influence on their members through their shared beliefs about teaching and through their norms for professional interaction” (p. 33). As described in the introduction, departments for VPE are responsible for the quality of the curriculum and instruction in the program. As the curriculum content is unique to the program, the members of the departments’ instructional team rely on each other to develop the pedagogical content knowledge (PCK) needed to teach the profession/trade well (Hoekstra and Newton 2017). PCK is used in tasks such as illustrating theory with engaging examples, lesson planning, addressing students’ questions, and establishing the pacing of content throughout the semester. PCK recognizes that certain subject matter requires content specific teaching methods and strategies. Based on the studies by Little (1990) it can be expected that how and whether instructors support one another in developing content specific teaching strategies, and in their teaching practice in general, is informed by their shared beliefs about teaching and their norms for professional interaction.

#### Autonomy

Autonomy has been widely regarded as a workplace condition favourable for professional learning (Kyndt 2016; Opfer and Pedder 2011). However, Clement and Vandenberghe (2000) identified a tension between autonomy and collegiality. At times autonomy in teacher’s work has been associated with resistance to a collective direction. In this respect Vangrieken et al. (2017) write: “equating autonomy to independence induces a negative attitude towards interdependent collaboration because teachers view this [interdependence] to be a threat to their autonomy” (p.303). Vangrieken et al. (2017) have further conceptualized and explored the relationship between autonomy and collaboration. Informed by the work of Deci and Ryan (1991) on psychological needs, they distinguish between a reactive and a reflective attitude towards autonomy. A *reactive autonomy attitude* reflects the traditional meaning of autonomy as freedom from the governance of others, an interpersonal process of resistance, and a focus on independence. A *reflective autonomy attitude* reflects the freedom to self-govern, an interpersonal process of personal choice, and a focus on interdependence. Within the school context collective pressures exist in terms of guidelines, norms, values, and influences. One might follow such influences from a place of obedience or coercion. However, a teacher using a reflective autonomy attitude would reflect on the influences and direction experienced, consider these influences for their value for student learning and make a personal choice to align their practice accordingly, rather than just doing what they are told. A reflective autonomy attitude would thus be more compatible with interdependent collaboration and collectively constructed course schedules, assessments etcetera. A reactive autonomy attitude could relate to less interdependent collaboration, with independent colleagues taking away from team conversations what suits them.

#### Perceptions of departmental leadership

For leadership in education to affect student outcomes, it should include a specific focus on student-learning. Thus, leadership for learning includes: (1) developing a shared vision and goals for student-learning; (2) promotion of teacher-learning; and (3) leading the educational program, including curriculum development and assessment of program quality (Hoekstra and Newton 2017; Hallinger 2011). The theoretical framework of leadership for learning is based on three leadership models: instructional, transformational, and shared leadership (Pietch et al. 2019). Promotion of teacher learning can happen through capacity building and transformational leadership: by direct mentoring, classroom observation and provision of feedback (Kyndt et al. 2016, 1133) or by creating a learning environment supportive of instructor learning (Hoekstra and Newton 2017). A shared vision and goals for student outcomes in a program provide both motivational power and clear communication and direction (Hallinger 2011). Additionally, Runhaar et al. (2010) found that perceptions of transformational leadership in VPE positively related to instructors’ reflection and willingness to solicit feedback. Shared leadership is focused on sharing the responsibility for improvement with the entire instructional team, thus actively promoting a sense of shared responsibility (Rechsteiner et al. 2022). As the effect of leadership actions on teacher learning is mediated by teachers’ perceptions of these actions (Louws et al. 2017; Rechsteiner et al. 2022), the present study focuses on instructors’ perceptions of how the leadership actions of their department chair impacts on their professional learning.

### Relationships between cultural conditions for learning

So, how do leadership for learning, shared norms and responsibility, autonomy and collaboration relate to each other? In post-secondary education the concept of academic freedom is used to describe the autonomy by instructors to make decisions on instruction and curriculum. While in some research-intensive university settings academic freedom may mean complete individual control over course content and delivery, institutes for VPE have curriculum that is created in collaboration and aligned to professional and trades standards. As the responsibility for the quality of instruction and curriculum in the program lies so clearly at the level of the VPE department, department chairs focused on leadership of learning would highlight that the quality of the program is the shared responsibility of the entire instructional team. Vanblaere and Devos (2018) found that the more teachers perceive their department head as a group-oriented leader, the more they report the presence of shared responsibility in the department. This perception of shared responsibility informs the way instructors work together. Oude Groote Beverborg et al. (2015) refer to goal interdependence as the extent of coordination needed to achieve collective goals, and they found a positive effect of goal interdependence on VET teachers’ learning. They also found an effect of transformational leadership on the extent to which VET teachers’ experienced goal interdependence. The distinction between reactive and reflective autonomy attitude can explain the role of autonomy in this interaction. Vangrieken et al. (2017) describe that a reactive autonomy attitude may be related to an aversion of teamwork, while a reflective autonomy attitude allows for individual choice to collaborate (p. 305). Based on these studies, it can be expected that when a department leader promotes a sense of shared responsibility, instructors with a reflective autonomy attitude would recognize the need for collaboration for the benefit of student learning and exert personal choice to work together (reflective autonomy attitude) towards instructional and curricular quality in their program, rather than focusing on independent work in isolation from colleagues. In the absence of a sense of a sense of shared responsibility, instructors might still seek out interaction or collaboration for mutual learning, but this would be in service of their own instructional practice. Finally, when the department leader is perceived as moving in a direction that is not in line with instructors’ norms and goals, instructors may adopt a reactive autonomy attitude and withdraw from collaboration at the department level. Such individual autonomy of staff members is identified as a barrier to school improvement (Imants et al. 2013, p. 329).

To explore how these conditions interact in practice, we employ a multiple case study approach to observe conditions for workplace learning in five different departments in institutes for VPE. The next section presents our research questions, followed by a description of our methods.

### Research questions

We aim to answer the following questions:

1. What structural and cultural conditions do instructors in departments for VPE report as enabling or inhibiting their professional learning?
2. Do departments differ in the ways they shape their workplace as an environment for instructor professional learning? And if so, how can these differences be explained?

It must be noted that data collection for this study took place prior to the Covid-19 pandemic and describes a pre-pandemic context and culture. During and since the Covid-19 pandemic a large portion of VPE programming was delivered online, with instructors working remotely. Anecdotally, the author has spoken with instructors who felt that working remotely has changed workplace culture and has contributed to feelings of isolation and lack of support. For administrators and department heads looking to foster conditions for instructor learning, strategies that worked when instructors shared physical spaces, may need to be adapted to maintain or recreate this culture in virtual spaces.

## Methods

Our study involves an exploratory multiple case study with the VPE department as the unit of analysis (Yin 1994). As we aimed to investigate instructor-learning in its natural context, the study follows a philosophy of naturalistic inquiry (Guba 1981) emphasizing participant’s own views as well as the context in which they express these views (Creswell 2005, 48).

### Recruitment, context, and ethics

Upon approval of our research ethics proposal, three publicly funded post-secondary institutes were approached for this study. To protect their identity, we use pseudonyms for research participants, institutes, and educational programs. Prairie College is a large institute for technical education, offering trades and diploma programs and two bachelor’s degrees. Mountain College is a teaching university with diploma and bachelor’s degree programs. River College is a community college with employment and upgrading programs as well as certificate and two-year diploma programs. Upon receiving further research ethics approval from the three institutes, deans were contacted to invite departments for inclusion in the study. We aimed to include a wide variety of educational programming. Five department chairs showed interest in participation. These five departments included provided trades, business, health technology, human service, and health science education respectively. Subsequently, instructors within each of these five departments were recruited to participate in the study and were requested to fill out an informed consent form. Recruitment happened through brief presentations at staff meetings and follow up emails forwarded by the chair to the various instructors. Instructors were assured that their chair would not be informed whether they volunteered or not. Interest from instructors in the departments varied. Instructors who did not volunteer for an interview often cited lack of time, either due to workload or personal commitments. Only four instructors from the business department volunteered; It is unclear whether this was due to lack of interest in the study or due to high workload. Exact response rates per department were not recorded, as the researchers were not provided with the exact numbers of instructors per department. From the other four departments more instructors volunteered than we needed for the study, so we randomly selected four or five instructors from average size departments (20–40 instructors) and six to eight instructors from large departments (> 40 instructors) to participate in an interview. This resulted in participation by 27 instructors.

### Sample, sites, and participants

Table 1 provides a general description of the five departments included in our study, as well as the age, gender, and years of teaching experience of the instructors in our sample. To protect the identity of our research participants, we chose not to list all participant characteristics by participant, because the unique combination of characteristics, such as age, gender, and years of experience, might allow colleagues to identify certain participants. Additionally, each participant is referred to with gender-neutral pseudonym to conceal their identity.

**Table 1:** General description of departments in study

College	Prairie college (Technical College)	Prairie college (Technical College)	Prairie college (Technical College)	Mountain college (Undergraduate University)	River college (2-year college)
Sector	Trade	Business	Health technology	Human service	Health service
Program	Apprenticeship	2-year diploma 4-year degree	2-year diploma	2-year diploma 4-year degree	2-year diploma
Pre-requisites	Employment in Trade + English & Math Grade 11	High school diploma	High school diploma	High school diploma	High school diploma
Number of instructors in department	> 40 instructors	20–40 instructors	10—20 full time	5 to 10 full time Some part time	> 40 instructors
Number and sex of instructors interviewed	8 (6 male; 2 female)	4 (1 male, 3 female)	5 (all female)	4 (1 male, 3 female)	6 (all female)
Age of instructors interviewed	2 31–40 yrs old 1 41–50 yrs old 3 51–60 yrs old 2 > 60 yrs old	1 < 31 yrs old 1 31–40 yrs old 1 41–50 yrs old 1 51–60 yrs old	1 < 30 yrs old 2 31–40 yrs old 1 51–60 yrs old 1 > 60 yrs old	1 41–50 yrs old 3 51–60 yrs old	1 31–40 yrs old 1 41–50 yrs old 2 51–60 yrs old 2 > 60 yrs old
Years of teaching experience of instructors interviewed	4 0–2 yrs 3 6–10 yrs 1 11–15 yrs	2 3–5 yrs 1 6–10 yrs 1 11–15 yrs	2 0–2 yrs 1 3–5 yrs 1 16–20 yrs 1 21–25 yrs	1 3–5 yrs 2 6–10 yrs 1 11–15 yrs	1 0–2 yrs 2 3–5 yrs 1 6–10 yrs 1 16–20 yrs 1 > 25 yrs
Chairs interviewed (Authors, 2017)	Blake (chair, 6 months) Drew (associate chair, 2 yrs)	Connor (Chair, 2 yrs)	Parker (chair, 3 yrs) Lane (associate chair, 2 yrs)	Sam (chair, 7 yrs)	Corey (chair, 3 weeks)
Trade/Profession specific values	Safety, efficiency	Accuracy, professionalism	Precision, detail, accuracy	Empathy, professionalism, ethics, respect	Patient safety; quality care
Trade/Profession specific regulations	Building code, Safety regulations	Integrity, due care, professionalism	Strict protocols; standards of practice	Much room for professional judgement, governmental directions and protocols	Standards of practice; code of ethics
Industry required PD for registered professionals, incl. instructors	None	Minimum 30 h of PD per year	Set learning goals, implement learning plan and report on progress	None	Set learning goals, implement learning plan and report on progress
Offices	Several office hubs on same campus	One office hub	Three main hubs on same campus	One office hub around a shared space	Several office hubs multiple campuses
Curriculum	Government mandated learning modules and course packs	Accredited by professional body; institutional quality guidelines	Accredited by professional body; institutional quality guidelines	Created by instructors; institutional quality guidelines	Accredited by professional body; institutional quality guidelines
Staff meetings	Once every two months; top down information sharing	Once every three months; Alternating (1) top-down information sharing OR (2) customized workshop	Monthly, combination of top-down information sharing and group discussion	Monthly, discussion of current practices, discussion of individual student issues; two-day spring retreat	Monthly, each meeting includes top down information sharing, presentations on relevant issues, some discussion
Collective digital space to share resources	Yes, well used	Yes	Yes, some use it	None	Yes, one digital space per course
Teaching assignments	Instructors rotate courses, and spend about three years teaching first year courses, then three years in 2nd year etc	By specialty	By specialty	By specialty	Groups of instructors by specialty and delivery mode (online, f2f, lab, clinical)

### Data collection and analysis

A semi-structured interview guide was developed based on an established method to study teacher learning and workplace conditions (Hoekstra et al. 2009). Participants were invited to one-on-one interviews by e-mail and were provided with the interview guide prior to the interviews. The interview focused on how learning is influenced by departmental conditions, with specific attention for perceived support from the department chair, collaboration, shared norms and goals, and autonomy. Interviews were conducted and audio recorded in mutually agreeable locations, and transcribed verbatim. To facilitate interpretation of the instructor interviews and to provide context and background, we also drew on chair interviews (See Hoekstra and Newton 2017), meeting observations, documents, and departmental and institutional websites.

### Interview analysis

Interview analysis focused on how the instructors experience the conditions they mention as relating to their learning. Interview segments that merely describe the field/trade, or how the department is organized were not coded if they did not contain information regarding the impact on instructor learning. In the analysis it became clear that one factor, for instance ‘performance management process’ could be experienced as supportive of learning by one instructor, but as unsupportive by another instructor. The work of Louws et al. (2017) inspired us to separate each code in an enabling and an inhibiting version. Hence, we coded interviews by workplace condition (Louws et al. 2017) and noted for each excerpt whether the condition was experienced as primarily enabling or inhibiting.

To establish reliability in coding, a colleague of the author was trained in the analysis method. The colleague and the author independently coded two transcripts and achieved 83% and 86% consistency in number of excerpts coded the same. Coding differences were discussed and agreed upon, and some code descriptions were more precisely formulated. Subsequently the author and colleague coded a third transcript independently and achieved 88% identical codes. The remainder of the transcripts were coded by either the author or the colleague and reviewed by the other. Inconsistencies were discussed and resolved. The final version of the code descriptions can be found in the Additional file 1.

Table 2 provides counts of the number of instructors per department who noted each condition as enabling or inhibiting, allowing comparison across departments (Miles and Huberman 1994).

**Table 2:** Number of instructors who mentioned a condition as enabling or inhibiting

Department	Trade N = 8		Business N = 4		Health Technology N = 6		Human Service N = 4		Health Service N = 6	
Code	Enabling	Inhibiting	Enabling	Inhibiting	Enabling	Inhibiting	Enabling	Inhibiting	Enabling	Inhibiting
***Structural Conditions***										
• Teaching schedule	2	4	1	–	2	–	–	–	–	4
• Workload	–	2	1	2	1	3	1	1	4	2
• Funding	6	–	–	–	1	–	3	–	6	–
• In–House PD	3	–	3	–	4	–	3	–	4	–
• External PD	7	–	2	–	4	–	3	–	2	–
• Learning materials	5	–	–	–	4	–	3	–	2	–
• Shared digital space	3	–	1	–	1	–	–	–	–	–
• Institutional student feedback on teaching survey present or lacking	–	3	–	2	–	–	3	–	3	–
• Office space	5	–	2	–	4	–	1	–	5	–
• Industry connections	2	–	1	–	5	–	4	–	4	–
• Continuing PD required for licencing	–	–	3	–	3	–	–	–	–	–
***Chair Support***										
• Chair as mentor	3	2	1	–	3	–	4	–	–	–
• Chair encourages PD	4	–	1	–	4	–	3	–	5	–
• Chair organizes PD	1	–	1	–	1	–	–	–	3	–
• Chair observes teaching	4	–	1	–	–	–	–	–	1	–
• Performance conversations supportive or not	4	3	2	1	3	1	1	–	2	3
***Culture***										
• Shared norms and goals	2	3	2	1	4	2	4	–	4	3
• Autonomy	8	5	2	3	4	2	4	1	5	3
• Mutual classroom observation	5	–	–	–	2	–	–	–	–	1
• Peer feedback	1	–	2	–	4	–	2	–	1	1
• Informal conversations	8	–	4	–	4	–	3	–	6	–
• Informal collaboration	6	–	1	2	2	–	3	–	4	1
• Regular meetings	4	–	1	1	2	1	4	–	3	1
• Reflective discussions	6	–	3	–	2	1	4	–	5	1
• Availability of colleagues as mentor	2	–	2	–	2	–	–	–	1	–

## Findings

### Structural conditions affecting instructor learning

Structural conditions within the workplace are conditions that follow from the way the work itself is organized. Before we discuss departmental conditions, it should be noted that at the organizational level, conditions for instructor learning appear to be favourable. Instructors within the participating institutes are entitled to a certain amount of professional development funding to attend conferences and courses. Additionally, each institute has an in-house department dedicated to providing courses and workshops and customized support for instructor learning. Departments have some funds at their discretion to purchase learning resources and other professional development (PD) opportunities for instructors. The institutes also have free internet access and an in-house library.

At the level of the department, we identified four structural conditions enabling learning and two conditions impeding learning.

*Student feedback* provided the most accessible learning opportunity for instructors and was common across departments and instructors in our study. Two institutes also offered a formalized process for formal feedback on instruction, through student evaluation forms.

*Job-rotation* occurred where instructors rotated courses or teaching different year levels. Hoekstra and Newton (2017) described how when assigned new (to them) courses to teach, instructors are prompted to develop new pedagogical content knowledge. The two-year and four-year programs in our study have instructors specialized by area of subject matter expertise and instructors may teach multiple year levels from the start. In the trades program, however, new instructors start teaching first year content only. Every few years they switch to teach content of the second, third, and the fourth/final year. A switch to teaching new content is preceded by the newly assigned instructor attending 10–20 lessons taught by colleagues in the content s/he is about to start teaching. In this trades program, instructor learning is thus built into the way instructors are assigned to courses.

*Coordinating work-placements* Several research participants reported tasks associated with arranging student placements. Through work-site visits, and coordinating activities, these instructors stay up to date with developments in the field.

*Continuing PD a licensing requirement* The licensing bodies of the health technology, health services, and business programs in our study require that instructors participate in a minimum number of hours of continuing professional development. From the interviews it was evident that this requirement clearly encourages instructors to engage in continuous professional learning.

*Teaching schedule* Instructors often mentioned high workload and teaching schedules to explain why they would not have time to innovate or attend PD events. While lack of time is often considered a structural factor impeding professional learning (Kyndt and Baert 2013; Lohman 2006) the data show that labelling a workplace factor as ‘lack of time’ obscures the various ways in which workload and teaching schedule impact instructor learning. For instance, in the Health, Human Services, and Business departments in our study instructors work 40–45 h on average during the semester, but semesters are 4 months each, and the business and human services instructors have reduced or no teaching load in May and June. This provides time for instructors to focus on course development and professional learning. Since trades instructors are in class 4–6 h a day, five days a week, for ten months, the teaching schedule in the study’s trades program prohibits participation in formal PD events that take 4 h or more. The chair allows substitute instructors, but in practice this is unrealistic, especially when groups of instructors want to attend the same PD opportunity. Similarly, the teaching schedule in the health services program also prohibits time away from the institute for professional development.

*Workload* Instructors in the trades department have an average workload of 30–35 h per week. This lower workload, along with a culture where instructors are expected to be on campus for 8 h a day, provides interested instructors time to observe colleagues teach and to engage in informal conversations about teaching and learning. Both health programs, however, report a much higher workload of 45–50 h per week during the semesters. Because of the nature of the programming, most instructors in these programs teach labs or online courses during May and June as well.

In summary, structural conditions supporting instructor learning at work include: (1) opportunities to collect student feedback; (2) rotation by course content or program year; (3) connecting with industry through student work-placement coordination; and (4) continuing PD requirements for licensing purposes. Structural conditions impeding instructor learning include: (5) high workload; and (6) teaching schedule.

### Departmental culture and leadership

While the structural conditions within each department are often outside of the direct control of individuals within the departments, the program culture and collaboration with the chair is enacted by the program teams. Table 2 shows that instructors from the five departments vary in the extent to which they describe conditions in their workplace as supportive of their learning. The human services department seems to have conditions most conducive of learning, while conditions in the business department are perceived as least conducive to instructor learning. The group norms, values, and ways of collaborating are interrelated with how the instructors view their chair’s response to and influence on these elements of the culture within the department. To illustrate these relationships, we provide three case descriptions in which we highlight how the cultural elements and perceptions of chair support are interrelated within the department and how they differ from the other departments. The three departments chosen allow us to illustrate the widest variety in practices, while staying within word limits.

#### Trades department

In the large trades department, instructors are organized in four groups around program content for one of the years of study. New instructors generally start teaching in year 1, and every few years move to teaching in a different year. This way instructors generally do not teach the same content for more than three years in a row. Year groups are led by an associate chair and meet about four times a year to discuss logistical issues, processes for student discipline, and absenteeism, and the development of common exams. In response to student complaints about content and rules not being consistent from one instructor to the next, there has been significant leadership effort over the years to implement consistent practices. The formal meetings observed by research team members were characterized by information sharing, mostly by the associate chair, with occasional discussion regarding select issues.

##### Shared norms and responsibility

Most instructors in the department agree with how Drew describes the goal of the program: ‘The end goal is for the students to attain their [trade] certification.’ While most instructors agree that this is the goal, there seems less agreement regarding how to best support students. For instance, Aiden said: ‘If I can instil something in [the students] where they're going to go out there and still want to learn weeks, months, and years after the course is over, then I think I've done my job.’ Having a shared understanding of this end goal seems to support instructor collaboration.

##### Collaboration

Within the department, groups of instructors are responsible for keeping course packs for the labs and exam bank questions accurate and up to date. To support instructor-rotation across courses in the various years, instructors are encouraged to observe other instructors’ classes prior to teaching the same content on their own. In addition, instructors in this trades department typically provide each other with teaching materials and convey what is important in terms of assisting student learning. River explained:

I enjoy sharing with the new guys. […] So, we'd be in the lab with them and show them how the different machines work and what they're looking for and what not to do and what's bad, what's good, so -- these are things you don't see on paper. These are things you get from working on the machines, right? And they always appreciate that.

In a sense, the requirement to rotate through the course content creates a sense of normalcy of continuous learning amongst the instructors in this department. Hollis: ‘[It’s] a department cultural thing where you share the stuff, […] there are other people coming up behind me who are just starting, then I try to do what people did for me when I started.’ Support for “the new guys” and “people coming up behind me” is reminiscent of the local apprenticeship system, where master trades people introduce apprentices to the work by showing them how the work ought to be done and sharing experiences for apprentices to learn from.

##### Autonomy

While there is an effort to create consistency, instructors feel that there is room within their own classroom to provide individual emphasis. For instance, Cory explains:

There's the [learning modules] … We have to teach off of those ... but if there’s other information … there are some interesting [trade related] things happening that aren't covered because they're brand new, and they didn't make it into the [learning modules] that I feel that it's worth going over with the students, and that's perfectly fine to do that.

Another example is Aiden recognizing a need for regulation of the industry: ‘I guess a part of me is pretty thankful that we have an industry that kind of says this is what's important to know and what's not important.’ The instructors appreciate the industry direction on curriculum and choose to align their practices accordingly.

In summary, the culture within the trades department in our study can be characterized as structurally-enabled informal collaboration among instructors in a context of externally provided curriculum. Despite expectations of delivering external curriculum, the environment empowers instructors to approach their teaching practice in individual ways, with an appreciation for leadership support for mutual learning amongst instructors. The way that support is readily offered to newer instructors mirrors the ways master tradespeople support apprentices in the field.

#### Business department

Reflecting a more individualized culture, the business department is course-focused. Apart from the first-year courses, instructors who taught the same course work in small groups to align pacing of content, quizzes, and exams. This work is usually led by a course coordinator.

*Shared norms and responsibility.* One of the instructors in our sample recently adopted the associate chair role. While s/he feels responsible for dealing with issues from all students in the program and states ‘I think that [the other instructors] feel a responsibility for their own students … only the [students] that they teach.’ A lack of shared norms for students’ learning sometimes leads to friction. Terry: ‘we do talk about a lot among ourselves, instructors. But we don't necessarily agree. Some instructors will do everything for the student and some instructors [think] that's too much babysitting.’ The lack of shared norms at times complicates collaboration.

*Collaboration.* Collaboration within the department includes coordination of course work. Marlin: ‘And in some courses it's very much a collaboration, and your opinions are, you feel like you can speak them. And in other courses it's like, I'm the coordinator, this is how I want the midterm to be, this is how the midterm is. So, it really depends.’ On an individual basis, instructors are ready to assist each other when asked. Dale: ‘the people [in our department], … you get that sense of ready to help. So, if that's the case, then you're more willing to do it too.’ Besides coordinating activities and one-on-one mutual support, collaboration within the department is limited. Terry: ‘I mean we don't really have formal department meetings to ask our input about … the program. The department meeting is more just about who can teach what –very general planning.’ The program chair organized several department wide meetings, where instructors were provided with information on new educational technologies and new institutional policies regarding instructor research opportunities. However, instructors did not reference these sessions as events that supported their learning.

Sam explains that while mutual support happens occasionally amongst instructors, s/he sees a parallel with how professionals in the business industry work and department culture: ‘[Out in industry], the more successful people are the ones who can just take things and figure them out. … Who won’t be like, ‘can somebody help me with this? I don’t get this.’ … Or find the resources on their own to figure it out’. Sam describes that in the field independent learning is highly valued:

From my experience in [name of field removed] firms, um, when you’re looking at performance review, you can be like, “oh, yeah. That person doesn’t know what’s going on. They’re asking all these people to help them; they’ve wasted so much time.” In [our field], you’re tracking every ten minutes of your time to make money, to be able to charge it out to your clients, so.

In the department itself these business instructors also seem to value independent learning: Instructors will support each other but only when asked, whereas in the trades department the support is readily offered by the more seasoned instructors.

##### Autonomy

The instructors in the business program are much less tied to a common curriculum than in the trades program. Dale: ‘in terms of autonomy and control […] you've got the syllabus which drills down and gives you autonomy in about 30 percent of your course evaluation and the way you're going to make the learning happen.’ The amount of autonomy instructors in the program experience is closely related to the courses they teach, the number of other instructors who teach that course, and the input they have in decisions around course content and materials. In this department, the tension between autonomy and a need for consistency seems resolved through the norm that 30 percent of course evaluation can be determined by the individual instructor.

In summary, instructors within the business department are organized by program courses, led by a course coordinator. While instructors do provide each other with mutual support, there is no department wide culture of continuous informal learning. This seems to mirror the culture in industry where self-reliance is valued. Instructors have control over a percentage of the assessment, which allows them the freedom to experiment and autonomy to decide on what elements of the course to emphasize, but there is not much input from instructors on department level issues.

#### Human services department

The team in the human services department is the smallest in our sample, with about 10–20 instructors. Their offices are all in the same office bank. While individual instructors have their own courses, the work is open to collegial scrutiny.

##### Shared norms and responsibility

In the interviews, the instructors from the human services department expressed that they share the same goals in terms of modelling best practices for the students. Alex describes:

[As a program] we have the professional side of what it is that you need to know out in the field. But we want to be creative. We want to be open. […] so, we convey that to our students that relationship is key when they're working out in the field. But then as instructors, the relationship is also key.

While the goals for students are the same, there is also an appreciation for the diverse ways instructors approach their teaching. Corbyn: ‘My role as an instructor is to help students learn who they are in this work’. Instructors highlight the importance of students developing their self-awareness and reflection, and respect for one another. Elsewhere in the interview s/he added ‘I think that our basic expectations are very similar. How we get there can be very different.’ Morgan said that ‘even with the differing views, we all agree in terms of the professionalism and the quality of education and the values of the program’.

##### Collaboration

There is a clear sense of collegiality. Corbyn describes that ‘It would be very rare that I would put a new assignment that's worth 30 percent in one of my courses without running it by a couple of my colleagues, right?’ Tristan adds: ‘We all have our course loads, so we're independent in that sense. [but] if anyone who has an issue with the course, it just gets thrown on the table. … You know, maybe you could do this and that.’ As Morgan explains, when issues with students arise, the team addresses these issues through reflective discussion:

Some of us, in our own sort of approaches or teaching styles or personal belief systems, might have different thoughts about how to address that issue. … So, we talk about those kinds of things, you know, are we helping or hindering this student?

The team thus engages in a shared reflection about how to support specific students. Morgan relates the culture in the program to the ethics of the professional practice the instructors used to work in:

I do believe that our particular group also in their own individual strengths and personalities and things that they bring from the field also bring a very similar ethic, you know, state of ethics in terms of—[…] we all come from practice backgrounds of helping people and wanting people to be the best that they can be in spite of, you know. And so that could be some of what we bring that gets really nurtured and supported and acknowledged and affirmed. Not only with each other, but I think the Chair has a huge piece in that as well.

The team participates in monthly meetings and a yearly two-day retreat organized by the chair, who is respected by the team members as a supportive mentor and coach.

##### Autonomy

While instructors are responsible for individual courses, instructors do not seem to give each other free reign. Alex explains how, subject to collective scrutiny, s/he needed to articulate the rationale for course changes:

But I do have a lot of autonomy because I teach a third-year course … And I've changed the face of that course. And certainly, there's been some critique of me doing that [I’ve had to] make my statements really clear [to] move forward in the way that I want to. So definitely there are some boundaries, but I always try to find a way to expand the boundaries a little bit.

In the human service department and the business department, the expectation is that professional learning is an individual instructor’s responsibility. However, in the human service department, the expectation is that instructors seek feedback from each other. For instructors in the human services department, a feeling of shared responsibility for student success, contributes to instructor’s openness to scrutiny by their peers. Little (1990) explains that in productive teams, ‘independent action is both constrained and enabled. Teachers open their intentions and practices to public examination, but in turn are credited for their knowledge, skill, and judgement.’ These elements of productive teams are clearly present in the human services department, while this sense of shared responsibility and peer scrutiny is largely absent in the business department.

In summary, in the human services department, instructors feel a shared responsibility for student success. There is an appreciation for differences in expertise and teaching approaches, combined with a sense of accountability to the program and to the profession. This work ethic aimed at nurturing each other’s strengths and accountability to the field is directly related to the values present in their previous work in the field and the profession taught in the program.

## Discussion

Our study explored the structural and cultural conditions VPE instructors report as enabling or inhibiting their professional learning, as well as any potential differences in conditions between various departments for VPE. The strengths of the study include the collection of both observational and interview data from 27 instructors in five departments for VPE, as well as documents. As such, our study complements the work of researchers who have conducted larger scale quantitative studies into instructor learning in VPE.

To a certain extent, the conditions for instructor professional learning in VPE in Western Canada confirm the findings on teacher learning in elementary and secondary schools and faculty development in higher education: there are resources for learning available, including the library and internet access; the use of student feedback is part of the job; and the organization is fairly flat with little opportunity for promotion. As in other types of educational organizations (Kyndt et al. 2016), our findings show that the lack of flexibility in instructor schedules can create barriers for collaborative professional learning and programmed professional development.

The findings also indicate that, similar to university settings and upper secondary education, the impact of structural and cultural conditions on instructor professional learning in VPE is mostly determined at the department level. This concurs with the findings of Knight and Trowler (2000) who looked at departmental practices in university settings. Both the study of Knight and Trowler and our own findings illustrate how departmental cultures and practices mediate the extent to which institutional provisions impact instructor learning. Our findings also concur with studies into department level practices in upper secondary education, that point to the educational department as organizational unit where collaborative cultures are most likely to form (e.g. Vanblaere and Devos 2018).

The case descriptions illustrate that culture for learning within the department can be influenced by the way the work is organized. In the trades department included in the study, instructor learning is normalized through job-rotation: every three or so years instructors change what year level they teach, eventually becoming all-round instructors. This requirement contributes to a culture of equality and mutual support and fosters instructor learning confirming findings from literature on human resource development (HRD) (Poell and Vanderkrogt 2014) that suggest job-rotation promotes learning at work.

While most of our findings confirm existing literature, we observed one phenomenon that to our knowledge has not been highlighted in existing literature on teacher learning. Due to the nature of the circumstances in VPE –instructors need to be licensed/experienced industry professionals and they become an instructor as their second career—we were able to observe how the socio-cultural background of the trade/profession shared by instructors influences the way instructors shape their workplace as a learning environment. The next section elaborates on this observation.

### Trade/profession as a socio-cultural background to workplace learning practices

Our case descriptions show that instructors within departments have specific ways of working together that can be related to the skills and strengths that are valued in the profession/trade that is taught in the department. In the human services department, we see values of the profession reflected in a high level of self-awareness amongst instructors, as well as an emphasis on learning through mutual reflection and relationship building. The trades department, which offers trades apprenticeship education, also has practices that mirror the profession, but in different ways than in human services. In the trades department, in learning how to teach, instructors display a learning model of apprenticeship: by observing more experienced colleagues, the newer instructors are basically learning the job of instructing and the applicable pedagogical content knowledge the same way apprentices in the field learn from their certified colleagues. Lastly, in the business department, instructors display a value of self-reliance and adherence to professional standards, mirroring values of the profession taught in this specific business program.

This mirroring of values from the profession/trade that instructors used to work within could be explained by the fact that instructors teaching the same trade/profession have all been socialized and have been successful in that trade/profession. In the VPE institute they continue to demonstrate these professional values in their day-to-day work as instructors. Billett (2011) explains that ‘the quality of individuals’ efforts to engage [with workplace learning opportunities] is influenced by their values, beliefs and socio-cultural background’ (67). Our findings suggest that groups of instructors who used to work in the same trade/industry before becoming an instructor draw on ‘elements of occupational culture’ (Coburn 2006, 345) in their collective sense-making.

The influence of elements of occupational culture in the way instructors shape their workplaces as learning environments, could explain the observations made by Andersson and Köpsén (2017) who found differences in the extent to which instructors from different industry sectors engaged in professional learning activities. Andersson and Köpsén (2017) found that instructors from the service industry had a higher uptake in certain learning activities than instructors from the construction industry. Further studies could look at the ways in which groups of instructors from different sectors draw on beliefs and values of the industry they used to work in, when deciding how and when to engage in workplace learning practices.

### Limitations

Our study has common limitations associated with qualitative studies, such as limited sample size and the fact that the study was conducted within one geographic region only. Additional limitations include a potential sampling bias, as instructors from the five departments volunteered to participate. Thankfully in four departments more instructors volunteered than we could accommodate, so for those departments we were able to do some random selection, slightly reducing the chance of sampling bias for those departments.

We were able to attend several department meetings in each department to corroborate and further contextualize interview data. However, the larger departments also have meetings in smaller sub-units, and instructors have informal meetings throughout their workday, which we were not able to attend. This means that our analysis of informal interaction and collaboration in smaller teams in the departments solely relied on interview data.

### Contributions and implications for research

Our case descriptions illustrate how cultural conditions for learning in the workplace are embedded in daily work and actively constructed in interaction. This is in line with studies on workplace learning that situate learning in the interaction between workplace affordances and individual agency (Billett 2004). Our findings also illustrate how various elements of departmental culture, including autonomy, collaboration, and shared responsibility interact. In the human services department, members of the instructional team contribute to professional learning by subjecting their course materials to collective scrutiny. This dependence on others is not, however, experienced as a lack of autonomy. We found the concept of a reflective autonomy attitude (Vangrieken et al. 2017) useful to describe how team members in the human services department exert collective agency: team members display a reflective autonomy attitude and actively choose to subject themselves to interdependent collaboration, as they recognize their collective responsibility to deliver quality education. In the business department some course coordinators deny their peers opportunities for mutual learning when they prescribe them the schedule, assignments, and collective exams for a course. In this business department there seems to be an emphasis on individual responsibility rather than shared responsibility, and instructors who do not agree with their course coordinator are still expected to follow what’s prescribed and might experience a lack of autonomy. The observation that workplace conditions are co-created in interaction highlights that workplace conditions cannot be understood by considering organizational factors alone. Further research could focus on how leadership impacts on instructors’ autonomy attitudes (Vangrieken et al. 2017) and how this relates to goal interdependence (Oude Groote Beverborg et al. 2015) and ultimately professional learning.

Secondly, we observed that the way instructors shape their workplace as a learning environment reflects the values and beliefs prevalent in the occupational culture of the trade/profession instructors used to work in. This observation could help explain differences in how instructors in the various industry sectors engage in professional learning (Andersson and Köpsén 2017). It seems that instructors’ beliefs about how one should be (or could be) learning-on-the-job are largely informed by the ways that they used to learn-on-the-job when they were still employed in their previous trade/profession. This influence of the previous trade/profession can be explained using the concept of sense-making (e.g. Coburn 2006). When making sense of their role as instructors and what it means to become better at one’s job, instructors draw on the cultural tools and constructs available to them. What is available to them is a mixture of previous experiences from their prior job, beliefs formed through those experiences, and the explanations and practices provided by their current colleagues in the VPE institute who also used to work in that same field. For instance, the trade program uses constructs of apprenticeship and cultural practices of ‘showing someone the ropes’, and ‘helping out the new guys’. When these trades instructors prepare themselves for a new task they go and observe a colleague to ‘see how it’s done’. More research is required to further understand differences in practices across many different types of industry groups, professional groups, and even multi-disciplinary groups (cf. Manuti et al. 2015), and the extent to which these differences can be explained by differences in beliefs. Part of this research could zoom in on the extent to which the instructional team in a department follows practices common in the original trade/profession versus practices promoted in the educational institute. Another part of the research could further explore the role of instructor beliefs about how one ought to learn at work. Researchers could extend the work by Thadani et al. (2015) who found that beliefs about whether teaching skills can change with effort were related to university faculty members’ interest in professional learning.

Future studies could also build on sense-making theory to further explore how groups of instructors draw on their collective occupational beliefs when they interact with leadership initiatives, for instance initiatives focused on establishing shared goals. Further studies could also focus on how department leaders could identify and possibly challenge instructors’ existing beliefs and practices related to how instructors learn at and for work. Finally, studies could focus on the ways in which structural and cultural conditions for workplace learning impact on each other.

### Implications for practice

Our findings illustrate how critical it is for instructional teams to successfully translate their cultural nuances in the profession to effective work-supportive environments for instructors. Leaders and team members in departments need to carefully consider cultural aspects, including beliefs, values, and common practices to create flexible options for learning and professional growth. Instructors may require support in recognizing and planning professional growth activities, for instance using professional growth plans (Beausaert et al. 2013) and explicit learning activities such as documenting experiences in a reflective journal (Viskovic 2005). It is critical for leadership, especially for chairs who come from the profession and are learning to lead, to create a sense of normalcy of continuous learning (cf. Hoekstra and Newton 2017). The rotation of courses is an example from the study that reinforces a structural condition enabling workplace learning at all levels of experience; and the findings indicate that the effectiveness of creating this sort of normalcy resides at the department level. A philosophy supporting this practice could be embedded at the institutional level but delivered appropriately at the department level—taking into consideration unique departmental needs but ensuring overall commitment to learning.

## Conclusion

Continuous (re)development of educational programming and professional learning by instructors is required for VPE institutes to remain relevant to society and industry. Our study illustrates how instructor learning is embedded in workplace practices. At the department level these practices are shaped through a process of sense-making that draws strongly on elements of the occupational culture taught in the program. Organizational support is warranted for the development of program leaders and to foster departmental conditions supportive of instructor learning.

### Supplementary Information

**Additional file 1: **Code descriptions and examples of excerpts from interviews.

## Data Availability

The datasets generated and analysed during the current study are not publicly available due to the need to protect the participants from being identified. Interview transcripts could be made available, after being redacted for identifying information, from the corresponding author on reasonable request.

## Abbreviations

PCK: Pedagogical content knowledge
PD: Professional development (Usually refers to one-time professional development workshops and courses)
HRD: Human resource development
VET: Vocational education and training
VPE: Vocational and professional education
