# Supplementary material for: Departmental conditions for professional learning of instructors in vocational and professional education
Source: Empir Res Vocat Educ Train. 2023 Oct 27;15(1):12. doi: 10.1186/s40461-023-00151-z (PMC10611886; doi:10.1186/s40461-023-00151-z)
Supplement: Supplementary file 1 — Additional file 1: Code descriptions and examples of excerpts from interviews. [file 40461_2023_151_MOESM1_ESM.docx]

**Departmental conditions for professional learning of instructors in vocational and professional education**

Additional Material. Code Descriptions and Examples Excerpts from Interviews

| **Code** | **Description** | **Examples from Interviews** |
| --- | --- | --- |
| Teaching Schedule allows for Participation in PD (enabling) | Able to participate in PD because either there was a break in the teaching schedule, or substitute instructors were arranged. | “They're always quite supportive for going to any courses you want to go.” [P1514, line 1071 – Health Services program] |
| Teaching Schedule does not allow for Participation in PD | PD took place at the time an instructor was scheduled to teach. | “I … did a one-day workshop. … But I don’t do enough of that….There’s not enough time, not enough people to replace me. I’m the only one who teaches this course, and if I’m sick, I don’t know what we’d do.” [P1565 lines 126-127 Health Services program]  “They have that big teaching and learning day, and they do it at the end of June, and then they do it again in August so everybody can go. And guess what? We're teaching full time both times.” [P1163, Trades, lines 1012-1016] |
| Time: Workload. Receives time for PD (enabling) | Instructor is scheduled a lesser workload that allows for PD. | “P1553: I’ve also taken a number of additional courses, one that was a three-day course on using technology” [P1553 lines 344-346 Health Services program] |
| Time: Workload. Heavy workload inhibits experimentation / research / improvement (inhibiting) | Instructor reports that s/he has too much work to engage in either formal PD or engage in activities aimed at improving teaching, teaching materials, assessments, etcetera. | “So really you're teaching five courses, and you're learning many different courses … And it's impacted how much energy people have to put into actually creating positive experiences for the students because you can't create if you don't have time.” [P1528 lines 474-483 Health Services program] |
| Funding available for PD (enabling) | Instructors mention there is funding available for them to participate in formal PD. | “We have $1,000 a year. There also is another $2,000 that you can get ‑‑ you can apply for when the ‑‑ like, two out of three years. […] If there's any money left over at the end of the year, you get this lovely email saying, Anybody got anything they want to do between now and June?” [P1417, Human Services program, lines 1191-1198] |
| In-house PD available (enabling) | Instructor mentions that the institute or program puts on PD events for staff/instructors. | “You know, there's a couple of things that – [in house centre for teaching and learning]. And they offer ‑‑ I get ‑‑ we get emails all the time, and they offer about where we can get together and different faculty from different ‑‑ or different professors from different faculty might present on, you know, something that they're ‑‑ have a particular expertise in. So I've attended a couple of those, which has been really interesting.” [P1420 Human Services program, lines 264-273] |
| External PD available (enabling) | Instructor mentions that s/he went to or intends to participate in PD put on by organizations other than their own institute. | “P1222: We also do industrial leave. So we get one or two days a year that we go offsite, and we go to a lab to keep us current and update in our field. We're encouraged to go to conferences and can even get some of the conference fees paid for and things like that.” [P1222, Health Technology program, lines 816-820] |
| Learning materials and resources available (enabling) | Instructor mentions that there are resources (journals, electronic resources, etcetera) available to learn from. | “I saw it in the [profession name] journal…” [P1565 line 109 Health Services program] |
| Access to shared digital space provided (enabling) | Instructors mention that within their department there is a digital space (e.g. share drive, USB stick, Online course in learning management system) where instructors can upload teaching materials to share with colleagues or instructors can access their colleagues’ teaching materials. | “They have, like, lots of documents that we provided on information, like, just on a computer stick.” [P1514 Health Services program, lines 136-138] |
| Institutional survey available (enabling) | Instructor mentions use of data from the institutional survey for student feedback on instruction for his/her own learning / professional development. | “[My institute] has a process of having formal evaluations online. And so it's not a huge response, but I do really talk to the students about why that's important and how it's helpful for students coming after them as well as for my own learning. And it's very important for me to hear, you know, what it was like for them.” [ P1420, Human Services program, lines 85-91] |
| Institutional survey unavailable (inhibiting) | Instructor mentions that no data are collected by means of an institutional survey for student feedback on instruction. | The online survey - [the college] is going through a new process now where they're trying to change that, so I ‑‑ it's no longer available. [P1146, Trades program, 259-260] |
| Office /work space (enabling) | Instructor mentions that conversations with colleagues happen in the office bank /work space. | Or we meet up and, you know, we've ‑‑ we ‑‑ some discussion starts up in the ‑‑ sort of the ‑‑ where the kitchen is or whatever, the food is. And then ‑‑ and you elaborate, and things happen. People talk. [1336, Business program 723-725] |
| Opportunities to connect with industry (enabling) | Has opportunities to talk to/interact with industry professionals. E.g. by working in industry part time, by engaging industry partners in curriculum development, or by assisting/accompanying students during their work-placements/clinicals. The instructor uses this experience to keep up with the trade/profession and have up to date content knowledge. | “I worked in the summer as a casual [health professional]. So kind of took those experiences and what I learned did on the floor, how I documented, how I took care of my clients.” [P1514, Health Services program, lines 298-300]  “Yeah, they're market‑driven. Like, what's new in health care. The new kind of tests. We stay close with industry. They tell us. I have ‑‑ you know, I'm less about the lab now and more about information. I have a little bit of a network. I stay informed as much as I can. [P1212, Health Technology program, lines 909-913] |
| Continuing PD required to stay registered (enabling) | Instructor needs to participate in certain continuing PD activities/events/workshops or maintain a learning portfolio in order to remain registered/ keep their license to practice in the trade/profession they teach. | “We have to, as part of our registration, … have to choose some goals, education—goals. … then, how are you going to—why is that goal important? And then each year, when I do my registration, … first, you have to go back and reflect on, so, ‘how did I meet that goal? What did I do?’ And plus, ‘what other educational professional development did I do?’” [P1553, Health Services program, lines 660-661] |

| **Experiences of CHAIR (or Associate Chair) Behaviour** | | |
| --- | --- | --- |
| Chair acts as mentor/advisor (enabling) | Instructor mentions that their chair advises or mentors instructors in the program - providing guidance, feedback; is a role model, etcetera. | “I think modelling and mentoring is another because I don't think there's a national or international conference that goes by that s/he (the chair) doesn't either facilitate at or co facilitate a workshop with one of the other instructors.” [P1420, Human Services program, lines 698-691]  “I think I get a lot of support from them. It's my Associate Chair in particular, like, I don't know, probably every day checks in. Like, How are you doing? Do you need anything? How are things going? Like, even those quick little questions.” [P1222, Health Technology, lines 915-919] |
| Chair encourages participation in PD (enabling) | Instructor mentions that the chair encourages / recommends / supports undertaking professional learning activities. | “He (the chair) also is quite … if there's a conference … they would send it out on email to say if you can go to this, this would be a great one to learn through.” [P1514, Health Services program, lines 1114-1118]  P1511: Yeah. Yeah, in the departmental team meeting, […] you present [what you learned at] the conference seminars that you went to. [P1511, Health Services program, lines 381-392] |
| Chair organizes PD opportunities (enabling) | The chair organizes work in such a way that there are learning opportunities for instructors; e.g. mentoring, PD sessions, PD in meetings. | “We have a new associate chair, and we meet with her, I think it’s once a month now, and she’s building in professional development into all of our meetings.” [P1585, Health Services program lines 400-406] |

| **Code** | **Description** | **Examples from Interviews** |
| --- | --- | --- |
| Chair observes teaching and provides feedback (enabling) | Instructor mentions that their chair observed their teaching and provided feedback. | “he also sat in on the ‑‑ a few of my lectures throughout that first year, and he would always give me feedback. And it was ‑‑ I always was impressed with how constructive and how generous he was with the feedback” [P1150, Trades program lines 181-187] |
| Performance conversations are supportive (enabling) | Instructor mentions that the performance management process as enacted in their department supports them in planning and/or evaluating their own learning. | P1171: P1171: But as time goes by, then there's more opportunities to do formal training and also encouragement to do that. So in the most recent performance evaluation […] so one of things that I want to do is, and this is sort of personal thing too, is do a little bit more for ESL students I've looked into doing a class in the spring that is teaching trades‑specific language so occupation‑specific language. [P1171, Trades program, lines 418-430] |
| Performance conversations are not supportive (inhibiting) | Instructors mention that their performance appraisal / performance management and/or review process does not have an impact on their PD or their learning. | “There's nothing in the performance planning process that I've been through that have encouraged me to take any … professional development.” [P1528, Health Services program lines 550-559] |

| **Experiences of Departmental Culture** | | |
| --- | --- | --- |
| Norms and goals for student learning are shared amongst instructors (enabling) | Instructor senses that s/he and his/her colleagues agree on norms and goals for what and how students need to learn. | “But I don't get the sense that even with that, the differing views, we're all in terms of the professionalism and the quality of education and the values of the program, that always stays the same.” [P1420 Human Services program, lines 565-568] |
| Norms and goals for student learning are not shared amongst colleagues (inhibiting) | Instructor senses that s/he and his/her colleagues do not agree on norms and goals for what and how students need to learn. | “Interviewer: And do you feel that you – that the other instructors in this department share that responsibility or that they feel that shared responsibility?  P1301: I think they feel a responsibility for their own students […]The ones they teach.” [P1301,. Business program, lines 700-706] |
| Autonomy over work (enabling) | Instructor feels that s/he can exercise their own judgement in how to do their job … and control over what they do and how they do it. | “To me, it forces me to learn and to do things that maybe I wouldn't normally do on myself, and so it sort of challenges you. And I think we learn a lot through challenges, you know, like, if I just keep things easy and keep doing it what I want to do, how I want to do it, I don't really improve or change anything. Whereas if I don't have control, for instance, like, the labs that I help in, I'm told what I'm doing in those. And so, you know, I have to adapt myself to them and sort of learn what somebody else is doing. […] And so for the lack of control, that was, I think, an important thing. But when you do have control, you know, I find that you focus on things you find interesting. And, you know, you put maybe a little bit more energy and effort into it because you just enjoy that more.” [P1222, Health Technology program, lines 620-643] |
| Lack of autonomy (inhibiting) | Instructor feels like s/he cannot exercise their own judgement, because they either have no control at all over these elements of their job, or they disagree with their colleagues | So, I feel sometimes we feel as instructors, a lot of clinical decisions are made, and the agenda’s being set already… But I—sometimes I feel we are powerless there, you know?” [P1514, Health Services program lines 860-864] |
| Culture of mutual classroom observation absent (inhibiting) | Instructors mention that it is uncommon for instructors to visit each others’ classes to observe and learn from how colleagues teach the material. | But it’s not a—it’s such a—I think it’s new: nobody does that. Like, no one has ever come to me, and said, “can I just come sit in your class and see how you teach?” [P1584, Health Services program, lines 362-370] |
| Culture of mutual classroom observation (enabling) | Instructors mention that it’s common for instructors to visit each others’ classes to observe and learn from how colleagues teach the material. | “It was two‑fold. I ‑‑ or multi‑fold actually. I went to see how the other instructors were delivering -- one on one at least, delivering the information. I used it as a way to refresh myself on material that I hadn't seen for five years. And then I also was watching how the students reacted to the different types of ‑‑ to the different instructors. Because there's so many different instructors in there at any one time, and each instructor seems to have a different teaching method.“ [P1150, Trades program, lines 157-165] |
| **Code** | **Description** | **Examples from Interviews** |
| Culture of peer feedback on teaching and/or materials present (enabling) | Instructor mentions it is quite common for colleagues in the department to either solicit or receive feedback from their peers on teaching or teaching materials, assignments, tests, etcetera. | “And being able to go to the former instructor of it and just pick their brain and be like, What did you do? How did it work? Can I try this? What do you think about this? And so I've got a really good collaboration with the other [subject matter] teacher.” [P1222, Health Technology program, lines 117-121] |
| Culture of feedback on teaching and/or materials from peers absent (inhibiting) | Instructor mentions it is not very common for colleagues in the department to either solicit or receive feedback from their peers on teaching, teaching materials, assignments, tests, etcetera. | “…because I found that what was lacking was that feedback from a professional; someone who had already been teaching.” [P1584, Health Services program, lines 66-68] |
| Colleagues available for informal conversations and sharing ideas (enabling) | Instructor mentions that colleagues are available for conversations to share ideas and that colleagues share resources. NOTE: Sharing ideas and sharing materials is different from collaboration. | “Or we might check in with each other, you know – ‘We've got this coming up. What are you doing for that? This is what I'm doing.’ And we might share ideas that way in terms of course how we're delivering that course.” [P1420 Human Services program, lines 141-145]  “and that's one thing about our department as a whole, is everybody shares the material, which is really helpful because you don't have to create everything right from the beginning.” [P1171, Trades program lines 197-200] |
| Colleagues available for informal collaboration (enabling) | When collaborating, instructors make something together or share a work load together; divide the work. | “So, we do collaborate a lot. There are times where we will—you know, there was something I didn’t finish off last week, so I’m going to have thirty minutes in the beginning, and then the rest of the class I’m leaving to my colleague, so, yes, we do collaborate in our team.” [P1565, Health Services program, lines 251-256]  You share drawings with others, they share them with you, and [together] you’ll just try to come up with better ways to get across a complex idea of how different parts of a machine work together, and so that’s one example. [P1146, Trades program, lines 182-198] |
| Colleagues are not available for informal collaboration (inhibiting) | Instructor mentions s/he is not able to collaborate with colleagues, either because they are too busy or not interested in collaboration. | “And even though there’s—so, there are […] teachers who teach in [different delivery modes], and the latest change, I tried to get their feedback, and really redesign the new assignment: nothing. They’re all too busy, right?” [P1584, Health Services program, lines 749-756] |
| Collaborates with colleagues during regular meetings (enabling) | Is part of a team that meets regularly for the purpose of collaboration . | “Yeah. I think as a team, we talk ‑‑ we probably talk more about students than most programs do, both individual and as a group. We have on ‑‑ at every staff meeting, we talk about students of concern. We also talk about students that are doing well, so we try not to get caught away from our own practice of strength‑based. [P1417 Human Services program, lines 202-208] |
| Experiences isolation when doing this work (inhibiting) | Instructor is isolated due to the nature of their work: either geographically isolated, or because they are the only one who work on this course/task. | “I’m fairly isolated, so I don’t feel a lot of collaboration. Because I’m the only one that teaches [this course] face-to-face. There isn’t another instructor that’s here that does that, so I don’t have another one that I have to go and talk with.” [P1584, Health Services program, lines 420-424] |
| Culture of reflective discussions (enabling) | Instructor mentions that it is common for groups of colleagues to engage in joint reflection on teaching situations, materials, assignments, etcetera. | “Uh, yeah, we had some discussions, mostly in regards to quizzes and if we should be making our own quiz, or if it’s better to have standardized quizzes, and what’s best for the students, and we ended up, um, deciding that we’re going to continue with the standardized quizzes, so.” [P1319, Business Program, lines 644-648] |
| Culture of reflective discussions absent (inhibiting) | Instructor mentions that it is not very common for groups of colleagues to engage in joint reflection on teaching situations, materials, assignments, etcetera. | There’s not a lot of interaction between instructors, unless you need to ask a specific question, or you’re saying “hi.” [P1319 Business Program, lines 519-521] |
| Colleagues available for classroom observation and/or as an informal mentor (enabling) | Instructor mentions that colleagues and/or the chair volunteer or, upon request, came to observe teaching and provided feedback; colleagues acted as informal mentors with teaching activity. | I experienced it when I got here, and I strongly maintain it. When a new instructor's here, I introduce myself, and anything I have is yours. And I'll attend your classrooms. You can attend mine. I've had instructors who come to they're new to fourth year. They have sat in through all my lectures for an entire [course] And I'm wide open to that. [P1128, Trades program 420-430] |
